# 4-{[5-(4-Chloro­phen­yl)-1-(4-fluoro­phen­yl)-1*H*-pyrazol-3-yl]carbon­yl}-*N*-(4-cyano­phen­yl)piperazine-1-carboxamide

**DOI:** 10.1107/S1600536810036159

**Published:** 2010-09-15

**Authors:** Wan-Sin Loh, Hoong-Kun Fun, R. Venkat Ragavan, V. Vijayakumar, M. Venkatesh

**Affiliations:** a X-ray Crystallography Unit, School of Physics, Universiti Sains Malaysia, 11800 USM, Penang, Malaysia; b Organic Chemistry Division, School of Advanced Sciences, VIT University, Vellore 632 014, India

## Abstract

In the title compound, C_28_H_22_ClFN_6_O_2_, the piperazine ring adopts a chair conformation and the least-squares plane through the four coplanar atoms forms dihedral angles of 69.37 (13) and 56.56 (12)°, respectively, with the pyrazole and cyano­phenyl rings. The dihedral angles formed between the pyrazole and the attached fluoro- and chloro­phenyl rings are 34.16 (10) and 73.27 (12)°, respectively. In the crystal, inter­molecular N—H⋯O, C—H⋯N and C—H⋯O hydrogen bonds link the mol­ecules into sheets parallel to the *ac* plane.

## Related literature

For background to pyrazole derivatives and their microbial activity, see: Ragavan *et al.* (2009 ▶, 2010 ▶). For the synthetic procedure, see: Ragavan *et al.* (2010 ▶). For ring conformations, see: Cremer & Pople (1975 ▶). For reference bond-length data, see: Allen *et al.* (1987 ▶). For related structures, see: Fun *et al.* (2010 ▶); Shahani *et al.* (2010 ▶). For the stability of the temperature controller used in the data collection, see: Cosier & Glazer (1986 ▶).
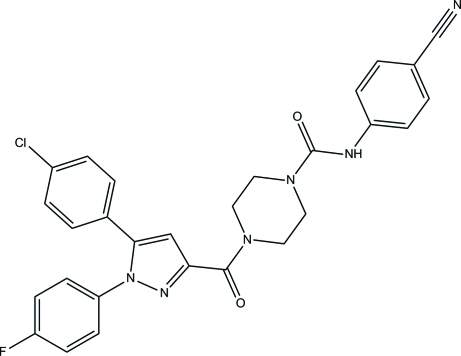


## Experimental

### 

#### Crystal data

C_28_H_22_ClFN_6_O_2_
*M* *_r_* = 528.97
Monoclinic, 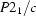

*a* = 9.9221 (3) Å
*b* = 21.3339 (7) Å
*c* = 12.7201 (4) Å
β = 111.629 (1)°
*V* = 2502.97 (14) Å^3^
*Z* = 4
Mo *K*α radiation
μ = 0.20 mm^−1^
*T* = 100 K
0.36 × 0.26 × 0.08 mm

#### Data collection

Bruker SMART APEXII CCD area-detector diffractometer
Absorption correction: multi-scan (*SADABS*; Bruker, 2009 ▶) *T*_min_ = 0.931, *T*_max_ = 0.985
19192 measured reflections
5660 independent reflections
4272 reflections with *I* > 2σ(*I*)
*R*_int_ = 0.037

#### Refinement

*R*[*F* ^2^ > 2σ(*F* ^2^)] = 0.054
*wR*(*F* ^2^) = 0.116
*S* = 1.07
5660 reflections
347 parameters
H atoms treated by a mixture of independent and constrained refinement
Δρ_max_ = 0.26 e Å^−3^
Δρ_min_ = −0.35 e Å^−3^

### 

Data collection: *APEX2* (Bruker, 2009 ▶); cell refinement: *SAINT* (Bruker, 2009 ▶); data reduction: *SAINT*; program(s) used to solve structure: *SHELXTL* (Sheldrick, 2008 ▶); program(s) used to refine structure: *SHELXTL*; molecular graphics: *SHELXTL*; software used to prepare material for publication: *SHELXTL* and *PLATON* (Spek, 2009 ▶).

## Supplementary Material

Crystal structure: contains datablocks global, I. DOI: 10.1107/S1600536810036159/wn2409sup1.cif

Structure factors: contains datablocks I. DOI: 10.1107/S1600536810036159/wn2409Isup2.hkl

Additional supplementary materials:  crystallographic information; 3D view; checkCIF report

## Figures and Tables

**Table 1:** Hydrogen-bond geometry (Å, °)

*D*—H⋯*A*	*D*—H	H⋯*A*	*D*⋯*A*	*D*—H⋯*A*
N5—H1*N*5⋯O1^i^	0.87 (3)	2.14 (3)	2.958 (3)	157 (2)
C2—H2*A*⋯N2^ii^	0.93	2.49	3.386 (3)	161
C4—H4*A*⋯O1^iii^	0.93	2.42	3.310 (3)	161
C7—H7*A*⋯O2^iv^	0.93	2.54	3.312 (3)	140

Symmetry codes: (i) 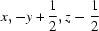; (ii) 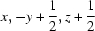; (iii) 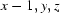; (iv) 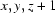.

**Fig. 1.**
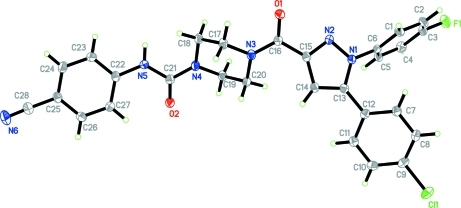
The molecular structure of the title compound, showing 50% probability displacement ellipsoids and the atom-numbering scheme. Hydrogen atoms are shown as spheres of arbitrary radius.

**Fig. 2.**
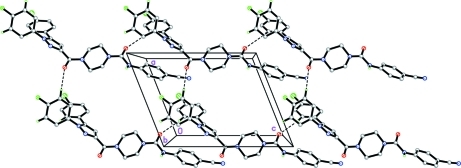
The crystal packing of the title compound, viewed along the b axis. H atoms not involved in the intermolecular interactions (dashed lines) have been omitted for clarity.

## supplementary crystallographic information

### Comment

The antibacterial and antifungal activities of azoles have been widely studied and some of them are used in clinical practice as antimicrobial agents. However, azole-resistant strains have led to the development of new antimicrobial compounds. In particular, pyrazole derivatives are extensively studied and used as antimicrobial agents. Pyrazoles form an important class of heterocyclic compound and many pyrazole derivatives are reported to have a broad spectrum of biological activities, such as anti-inflammatory, antifungal, herbicidal, antitumor, cytotoxic and antiviral activities; they are also used in molecular modelling. Pyrazole derivatives also act as anti-angiogenic agents, A3 adenosine receptor antagonists, neuropeptide YY5 receptor antagonists as well as kinase inhibitor for the treatment of type 2 diabetes, hyperlipidemia, obesity and thrombopiotinmimetics. Recently urea derivatives of pyrazoles have been reported as potent inhibitors of p38 kinase. Since the high electronegativity of halogens (particularly chlorine and fluorine) in the aromatic part of drug molecules play an important role in enhancing their biological activity, we are interested in compounds having 4-fluoro- or 4-chloro-substitution in 1,5-diaryl pyrazoles. The background to pyrazole derivatives and their microbial activities habe been reported in reccent years (Ragavan *et al.*, 2009, 2010). The crystal structure of the title compound is reported here.

In the title compound (Fig. 1) the piperazine ring adopts a chair conformation with puckering parameters (Cremer & Pople, 1975) of Q = 0.540 (2) Å, Θ = 1.3 (2)°, φ = 235 (21)° and the plane through the coplanar atoms (N4/C19/N3/C17) forms dihedral angles of 69.37 (13) and 56.56 (12)°, respectively, with the pyrazole and cyanophenyl rings. The dihedral angles formed between the pyrazole and attached fluoro- and chlorophenyl rings are 34.16 (10) and 73.27 (12)°, respectively. Bond lengths (Allen *et al.*, 1987) and angles are within the normal ranges and are comparable to those in related crystal structures (Fun *et al.*, 2010; Shahani *et al.*, 2010).

In the crystal packing (Fig. 2), intermolecular N5—H1N5···O1, C2—H2A···N2, C4—H4A···O1 and C7—H7A···O2 hydrogen bonds (Table 1) link the molecules into two-dimensional sheets parallel to the *ac* plane.

### Experimental

The compound has been synthesized using a method reported  in the literature (Ragavan *et al.*, 2010) and recrystallized using a 1:1 mixture of ethanol-chloroform. Yield = 77%. *M. p.* = 485.3–486 K.

### Refinement

Atom H1N5 was located in a difference Fourier map and was refined freely [N—H = 0.87 (3) Å]. The remaining H atoms were positioned geometrically [C—H = 0.93 or 0.97 Å] and were refined using a riding model, with *U*_iso_(H) = 1.2 *U*_eq_(C).

### Crystal data

**Table:**

C_28_H_22_ClFN_6_O_2_	*F*(000) = 1096
*M**_r_* = 528.97	*D*_x_ = 1.404 Mg m^−^^3^
Monoclinic, *P*2_1_/*c*	Mo *K*α radiation, λ = 0.71073 Å
Hall symbol: -P 2ybc	Cell parameters from 5338 reflections
*a* = 9.9221 (3) Å	θ = 2.4–27.3°
*b* = 21.3339 (7) Å	µ = 0.20 mm^−^^1^
*c* = 12.7201 (4) Å	*T* = 100 K
β = 111.629 (1)°	Plate, colourless
*V* = 2502.97 (14) Å^3^	0.36 × 0.26 × 0.08 mm
*Z* = 4	

### Data collection

**Table:**

Bruker SMART APEXII CCD area-detector diffractometer	5660 independent reflections
Radiation source: fine-focus sealed tube	4272 reflections with *I* > 2σ(*I*)
graphite	*R*_int_ = 0.037
φ and ω scans	θ_max_ = 27.4°, θ_min_ = 1.9°
Absorption correction: multi-scan (*SADABS*; Bruker, 2009)	*h* = −12→11
*T*_min_ = 0.931, *T*_max_ = 0.985	*k* = −27→24
19192 measured reflections	*l* = −14→16

### Refinement

**Table:**

Refinement on *F*^2^	Primary atom site location: structure-invariant direct methods
Least-squares matrix: full	Secondary atom site location: difference Fourier map
*R*[*F*^2^ > 2σ(*F*^2^)] = 0.054	Hydrogen site location: inferred from neighbouring sites
*wR*(*F*^2^) = 0.116	H atoms treated by a mixture of independent and constrained refinement
*S* = 1.07	*w* = 1/[σ^2^(*F*_o_^2^) + (0.0294*P*)^2^ + 2.8225*P*] where *P* = (*F*_o_^2^ + 2*F*_c_^2^)/3
5660 reflections	(Δ/σ)_max_ < 0.001
347 parameters	Δρ_max_ = 0.26 e Å^−^^3^
0 restraints	Δρ_min_ = −0.35 e Å^−^^3^

### Special details

**Table:**

Experimental. The crystal was placed in the cold stream of an Oxford Cryosystems Cobra open-flow nitrogen cryostat (Cosier & Glazer, 1986) operating at 100.0 (1) K.
Geometry. All e.s.d.'s (except the e.s.d. in the dihedral angle between two l.s. planes) are estimated using the full covariance matrix. The cell e.s.d.'s are taken into account individually in the estimation of e.s.d.'s in distances, angles and torsion angles; correlations between e.s.d.'s in cell parameters are only used when they are defined by crystal symmetry. An approximate (isotropic) treatment of cell e.s.d.'s is used for estimating e.s.d.'s involving l.s. planes.
Refinement. Refinement of *F*^2^ against ALL reflections. The weighted *R*-factor *wR* and goodness of fit *S* are based on *F*^2^, conventional *R*-factors *R* are based on *F*, with *F* set to zero for negative *F*^2^. The threshold expression of *F*^2^ > σ(*F*^2^) is used only for calculating *R*-factors(gt) *etc*. and is not relevant to the choice of reflections for refinement. *R*-factors based on *F*^2^ are statistically about twice as large as those based on *F*, and *R*- factors based on ALL data will be even larger.

### Fractional atomic coordinates and isotropic or equivalent isotropic displacement parameters (Å^2^)

**Table:**

	*x*	*y*	*z*	*U*_iso_*/*U*_eq_	
Cl1	0.47534 (7)	0.60803 (3)	0.78980 (5)	0.03029 (16)	
F1	0.48142 (15)	0.22562 (7)	0.89619 (12)	0.0304 (3)	
N1	0.81146 (19)	0.34593 (9)	0.69103 (14)	0.0161 (4)	
N2	0.89747 (19)	0.31063 (9)	0.65254 (14)	0.0164 (4)	
N3	1.04762 (19)	0.34908 (9)	0.44459 (14)	0.0174 (4)	
N4	1.0136 (2)	0.35012 (9)	0.21385 (15)	0.0203 (4)	
N5	1.1177 (2)	0.35113 (10)	0.07780 (16)	0.0194 (4)	
N6	1.2635 (2)	0.45747 (11)	−0.37105 (17)	0.0335 (5)	
O1	1.14153 (16)	0.28689 (7)	0.59902 (12)	0.0193 (3)	
O2	0.91589 (17)	0.41027 (8)	0.05706 (13)	0.0236 (4)	
C1	0.7918 (2)	0.29390 (11)	0.85563 (18)	0.0197 (5)	
H1A	0.8905	0.2999	0.8948	0.024*	
C2	0.7083 (2)	0.26399 (11)	0.90742 (19)	0.0220 (5)	
H2A	0.7493	0.2503	0.9819	0.026*	
C3	0.5637 (2)	0.25525 (11)	0.84547 (19)	0.0216 (5)	
C4	0.4962 (2)	0.27504 (11)	0.73509 (19)	0.0228 (5)	
H4A	0.3978	0.2681	0.6959	0.027*	
C5	0.5800 (2)	0.30562 (11)	0.68465 (18)	0.0203 (5)	
H5A	0.5380	0.3200	0.6106	0.024*	
C6	0.7266 (2)	0.31466 (10)	0.74509 (18)	0.0163 (5)	
C7	0.7134 (2)	0.45597 (11)	0.79798 (18)	0.0209 (5)	
H7A	0.7593	0.4246	0.8494	0.025*	
C8	0.6372 (2)	0.50256 (11)	0.82822 (19)	0.0227 (5)	
H8A	0.6303	0.5021	0.8992	0.027*	
C9	0.5715 (2)	0.54962 (11)	0.75187 (19)	0.0213 (5)	
C10	0.5806 (2)	0.55132 (11)	0.64628 (19)	0.0218 (5)	
H10A	0.5365	0.5835	0.5961	0.026*	
C11	0.6562 (2)	0.50443 (11)	0.61567 (18)	0.0194 (5)	
H11A	0.6629	0.5054	0.5447	0.023*	
C12	0.7220 (2)	0.45589 (11)	0.69021 (18)	0.0177 (5)	
C13	0.8020 (2)	0.40700 (11)	0.65574 (17)	0.0166 (5)	
C14	0.8854 (2)	0.41079 (11)	0.58986 (17)	0.0176 (5)	
H14A	0.9007	0.4460	0.5526	0.021*	
C15	0.9423 (2)	0.35043 (11)	0.59080 (17)	0.0163 (5)	
C16	1.0502 (2)	0.32613 (10)	0.54447 (17)	0.0157 (4)	
C17	1.1727 (2)	0.33981 (11)	0.41143 (18)	0.0192 (5)	
H17A	1.2416	0.3120	0.4652	0.023*	
H17B	1.2204	0.3797	0.4134	0.023*	
C18	1.1283 (2)	0.31192 (11)	0.29329 (17)	0.0205 (5)	
H18A	1.2112	0.3107	0.2703	0.025*	
H18B	1.0939	0.2694	0.2934	0.025*	
C19	0.8884 (2)	0.35880 (11)	0.24632 (18)	0.0202 (5)	
H19A	0.8419	0.3187	0.2449	0.024*	
H19B	0.8189	0.3861	0.1921	0.024*	
C20	0.9325 (2)	0.38715 (11)	0.36402 (17)	0.0195 (5)	
H20A	0.9668	0.4297	0.3634	0.023*	
H20B	0.8493	0.3886	0.3867	0.023*	
C21	1.0089 (2)	0.37302 (11)	0.11281 (18)	0.0179 (5)	
C22	1.1497 (2)	0.37577 (11)	−0.01271 (17)	0.0185 (5)	
C23	1.2352 (3)	0.33979 (11)	−0.05514 (19)	0.0235 (5)	
H23A	1.2688	0.3009	−0.0230	0.028*	
C24	1.2707 (3)	0.36094 (12)	−0.1441 (2)	0.0261 (5)	
H24A	1.3268	0.3361	−0.1722	0.031*	
C25	1.2229 (2)	0.41924 (11)	−0.19194 (18)	0.0204 (5)	
C26	1.1417 (3)	0.45604 (12)	−0.1474 (2)	0.0255 (5)	
H26A	1.1115	0.4956	−0.1775	0.031*	
C27	1.1050 (3)	0.43481 (11)	−0.0591 (2)	0.0247 (5)	
H27A	1.0502	0.4600	−0.0304	0.030*	
C28	1.2494 (3)	0.44038 (12)	−0.29033 (19)	0.0242 (5)	
H1N5	1.148 (3)	0.3133 (14)	0.098 (2)	0.033 (8)*	

### Atomic displacement parameters (Å^2^)

**Table:**

	*U*^11^	*U*^22^	*U*^33^	*U*^12^	*U*^13^	*U*^23^
Cl1	0.0278 (3)	0.0295 (4)	0.0347 (3)	0.0067 (3)	0.0129 (3)	−0.0071 (3)
F1	0.0281 (8)	0.0372 (9)	0.0311 (8)	−0.0038 (7)	0.0170 (6)	0.0075 (7)
N1	0.0167 (9)	0.0172 (10)	0.0156 (9)	0.0006 (8)	0.0074 (7)	0.0003 (8)
N2	0.0161 (9)	0.0184 (10)	0.0156 (9)	−0.0001 (8)	0.0069 (7)	−0.0017 (8)
N3	0.0154 (9)	0.0217 (10)	0.0151 (9)	0.0004 (8)	0.0056 (7)	0.0006 (8)
N4	0.0192 (10)	0.0268 (11)	0.0163 (9)	0.0060 (8)	0.0081 (7)	0.0040 (8)
N5	0.0244 (11)	0.0174 (11)	0.0190 (9)	0.0042 (9)	0.0110 (8)	0.0024 (8)
N6	0.0404 (13)	0.0391 (14)	0.0255 (11)	−0.0110 (11)	0.0175 (10)	−0.0008 (10)
O1	0.0193 (8)	0.0207 (9)	0.0175 (8)	0.0024 (7)	0.0063 (6)	0.0022 (7)
O2	0.0264 (9)	0.0278 (10)	0.0190 (8)	0.0088 (7)	0.0111 (7)	0.0068 (7)
C1	0.0176 (11)	0.0217 (13)	0.0187 (11)	0.0025 (9)	0.0055 (9)	0.0024 (9)
C2	0.0236 (12)	0.0258 (14)	0.0167 (11)	0.0039 (10)	0.0076 (9)	0.0062 (10)
C3	0.0255 (13)	0.0209 (13)	0.0237 (11)	−0.0025 (10)	0.0153 (10)	0.0007 (10)
C4	0.0152 (11)	0.0278 (14)	0.0241 (12)	−0.0018 (10)	0.0058 (9)	−0.0017 (10)
C5	0.0214 (12)	0.0241 (13)	0.0148 (10)	0.0016 (10)	0.0060 (9)	0.0011 (10)
C6	0.0191 (11)	0.0147 (11)	0.0176 (10)	0.0008 (9)	0.0096 (9)	−0.0004 (9)
C7	0.0233 (12)	0.0220 (13)	0.0165 (10)	0.0017 (10)	0.0062 (9)	−0.0002 (10)
C8	0.0253 (12)	0.0255 (13)	0.0190 (11)	−0.0005 (10)	0.0102 (9)	−0.0040 (10)
C9	0.0162 (11)	0.0194 (12)	0.0279 (12)	−0.0015 (9)	0.0076 (9)	−0.0064 (10)
C10	0.0188 (12)	0.0188 (13)	0.0262 (12)	0.0011 (10)	0.0063 (9)	0.0028 (10)
C11	0.0179 (11)	0.0211 (13)	0.0189 (10)	−0.0033 (9)	0.0064 (8)	−0.0005 (9)
C12	0.0167 (11)	0.0170 (12)	0.0198 (10)	−0.0033 (9)	0.0073 (8)	−0.0043 (9)
C13	0.0172 (11)	0.0173 (12)	0.0131 (10)	−0.0010 (9)	0.0030 (8)	−0.0008 (9)
C14	0.0192 (11)	0.0181 (12)	0.0163 (10)	−0.0030 (9)	0.0076 (9)	−0.0009 (9)
C15	0.0167 (11)	0.0185 (12)	0.0124 (10)	−0.0027 (9)	0.0038 (8)	−0.0016 (9)
C16	0.0160 (11)	0.0164 (12)	0.0139 (10)	−0.0046 (9)	0.0045 (8)	−0.0041 (9)
C17	0.0164 (11)	0.0245 (13)	0.0170 (10)	−0.0016 (9)	0.0066 (9)	0.0003 (10)
C18	0.0201 (12)	0.0274 (13)	0.0154 (10)	0.0049 (10)	0.0083 (9)	0.0025 (10)
C19	0.0177 (11)	0.0265 (13)	0.0165 (10)	0.0026 (10)	0.0064 (9)	0.0027 (10)
C20	0.0192 (11)	0.0226 (13)	0.0176 (10)	0.0035 (10)	0.0080 (9)	0.0020 (9)
C21	0.0185 (11)	0.0184 (12)	0.0164 (10)	−0.0021 (9)	0.0060 (9)	−0.0023 (9)
C22	0.0207 (12)	0.0224 (13)	0.0119 (10)	−0.0030 (10)	0.0056 (8)	−0.0028 (9)
C23	0.0296 (13)	0.0205 (13)	0.0227 (11)	0.0054 (10)	0.0124 (10)	0.0034 (10)
C24	0.0289 (13)	0.0304 (15)	0.0233 (12)	0.0052 (11)	0.0146 (10)	−0.0003 (11)
C25	0.0220 (12)	0.0234 (13)	0.0161 (10)	−0.0040 (10)	0.0076 (9)	−0.0015 (9)
C26	0.0349 (14)	0.0199 (13)	0.0250 (12)	0.0020 (11)	0.0150 (10)	0.0028 (10)
C27	0.0339 (14)	0.0200 (13)	0.0259 (12)	0.0034 (11)	0.0179 (11)	0.0004 (10)
C28	0.0252 (13)	0.0265 (14)	0.0203 (12)	−0.0063 (10)	0.0077 (10)	−0.0034 (10)

### Geometric parameters (Å, °)

**Table:**

Cl1—C9	1.742 (2)	C8—H8A	0.9300
F1—C3	1.367 (2)	C9—C10	1.379 (3)
N1—N2	1.358 (2)	C10—C11	1.389 (3)
N1—C13	1.370 (3)	C10—H10A	0.9300
N1—C6	1.433 (3)	C11—C12	1.393 (3)
N2—C15	1.339 (3)	C11—H11A	0.9300
N3—C16	1.353 (3)	C12—C13	1.472 (3)
N3—C17	1.464 (3)	C13—C14	1.380 (3)
N3—C20	1.467 (3)	C14—C15	1.405 (3)
N4—C21	1.360 (3)	C14—H14A	0.9300
N4—C19	1.458 (3)	C15—C16	1.492 (3)
N4—C18	1.460 (3)	C17—C18	1.523 (3)
N5—C21	1.392 (3)	C17—H17A	0.9700
N5—C22	1.404 (3)	C17—H17B	0.9700
N5—H1N5	0.87 (3)	C18—H18A	0.9700
N6—C28	1.146 (3)	C18—H18B	0.9700
O1—C16	1.241 (3)	C19—C20	1.522 (3)
O2—C21	1.226 (3)	C19—H19A	0.9700
C1—C6	1.386 (3)	C19—H19B	0.9700
C1—C2	1.389 (3)	C20—H20A	0.9700
C1—H1A	0.9300	C20—H20B	0.9700
C2—C3	1.372 (3)	C22—C23	1.392 (3)
C2—H2A	0.9300	C22—C27	1.392 (3)
C3—C4	1.380 (3)	C23—C24	1.380 (3)
C4—C5	1.386 (3)	C23—H23A	0.9300
C4—H4A	0.9300	C24—C25	1.389 (3)
C5—C6	1.386 (3)	C24—H24A	0.9300
C5—H5A	0.9300	C25—C26	1.387 (3)
C7—C8	1.387 (3)	C25—C28	1.443 (3)
C7—C12	1.404 (3)	C26—C27	1.378 (3)
C7—H7A	0.9300	C26—H26A	0.9300
C8—C9	1.382 (3)	C27—H27A	0.9300
			
N2—N1—C13	112.69 (17)	C15—C14—H14A	127.2
N2—N1—C6	118.25 (17)	N2—C15—C14	111.51 (18)
C13—N1—C6	128.21 (18)	N2—C15—C16	116.95 (19)
C15—N2—N1	104.40 (17)	C14—C15—C16	131.30 (19)
C16—N3—C17	119.79 (18)	O1—C16—N3	121.78 (19)
C16—N3—C20	126.63 (18)	O1—C16—C15	119.71 (18)
C17—N3—C20	113.48 (17)	N3—C16—C15	118.49 (19)
C21—N4—C19	119.10 (18)	N3—C17—C18	111.66 (17)
C21—N4—C18	126.94 (18)	N3—C17—H17A	109.3
C19—N4—C18	113.76 (17)	C18—C17—H17A	109.3
C21—N5—C22	125.0 (2)	N3—C17—H17B	109.3
C21—N5—H1N5	116.6 (18)	C18—C17—H17B	109.3
C22—N5—H1N5	115.4 (18)	H17A—C17—H17B	108.0
C6—C1—C2	119.4 (2)	N4—C18—C17	109.47 (18)
C6—C1—H1A	120.3	N4—C18—H18A	109.8
C2—C1—H1A	120.3	C17—C18—H18A	109.8
C3—C2—C1	118.1 (2)	N4—C18—H18B	109.8
C3—C2—H2A	121.0	C17—C18—H18B	109.8
C1—C2—H2A	121.0	H18A—C18—H18B	108.2
F1—C3—C2	118.2 (2)	N4—C19—C20	111.31 (18)
F1—C3—C4	118.1 (2)	N4—C19—H19A	109.4
C2—C3—C4	123.7 (2)	C20—C19—H19A	109.4
C3—C4—C5	117.8 (2)	N4—C19—H19B	109.4
C3—C4—H4A	121.1	C20—C19—H19B	109.4
C5—C4—H4A	121.1	H19A—C19—H19B	108.0
C6—C5—C4	119.7 (2)	N3—C20—C19	109.55 (18)
C6—C5—H5A	120.2	N3—C20—H20A	109.8
C4—C5—H5A	120.2	C19—C20—H20A	109.8
C5—C6—C1	121.3 (2)	N3—C20—H20B	109.8
C5—C6—N1	118.75 (19)	C19—C20—H20B	109.8
C1—C6—N1	119.91 (19)	H20A—C20—H20B	108.2
C8—C7—C12	120.5 (2)	O2—C21—N4	122.4 (2)
C8—C7—H7A	119.8	O2—C21—N5	122.7 (2)
C12—C7—H7A	119.8	N4—C21—N5	114.94 (19)
C9—C8—C7	119.3 (2)	C23—C22—C27	118.6 (2)
C9—C8—H8A	120.3	C23—C22—N5	117.7 (2)
C7—C8—H8A	120.3	C27—C22—N5	123.6 (2)
C10—C9—C8	121.4 (2)	C24—C23—C22	121.0 (2)
C10—C9—Cl1	119.32 (18)	C24—C23—H23A	119.5
C8—C9—Cl1	119.31 (18)	C22—C23—H23A	119.5
C9—C10—C11	119.3 (2)	C23—C24—C25	120.2 (2)
C9—C10—H10A	120.3	C23—C24—H24A	119.9
C11—C10—H10A	120.3	C25—C24—H24A	119.9
C10—C11—C12	120.7 (2)	C26—C25—C24	119.0 (2)
C10—C11—H11A	119.7	C26—C25—C28	119.8 (2)
C12—C11—H11A	119.7	C24—C25—C28	121.2 (2)
C11—C12—C7	118.8 (2)	C27—C26—C25	121.0 (2)
C11—C12—C13	119.52 (19)	C27—C26—H26A	119.5
C7—C12—C13	121.7 (2)	C25—C26—H26A	119.5
N1—C13—C14	105.71 (19)	C26—C27—C22	120.3 (2)
N1—C13—C12	123.74 (19)	C26—C27—H27A	119.9
C14—C13—C12	130.5 (2)	C22—C27—H27A	119.9
C13—C14—C15	105.69 (19)	N6—C28—C25	176.8 (3)
C13—C14—H14A	127.2		
			
C13—N1—N2—C15	−0.5 (2)	C13—C14—C15—N2	0.4 (2)
C6—N1—N2—C15	169.86 (18)	C13—C14—C15—C16	−173.7 (2)
C6—C1—C2—C3	1.1 (3)	C17—N3—C16—O1	−14.7 (3)
C1—C2—C3—F1	179.8 (2)	C20—N3—C16—O1	169.2 (2)
C1—C2—C3—C4	−0.7 (4)	C17—N3—C16—C15	163.51 (19)
F1—C3—C4—C5	179.3 (2)	C20—N3—C16—C15	−12.6 (3)
C2—C3—C4—C5	−0.2 (4)	N2—C15—C16—O1	−33.1 (3)
C3—C4—C5—C6	0.7 (3)	C14—C15—C16—O1	140.7 (2)
C4—C5—C6—C1	−0.2 (3)	N2—C15—C16—N3	148.6 (2)
C4—C5—C6—N1	179.5 (2)	C14—C15—C16—N3	−37.6 (3)
C2—C1—C6—C5	−0.7 (3)	C16—N3—C17—C18	128.3 (2)
C2—C1—C6—N1	179.6 (2)	C20—N3—C17—C18	−55.1 (3)
N2—N1—C6—C5	−102.4 (2)	C21—N4—C18—C17	130.4 (2)
C13—N1—C6—C5	66.2 (3)	C19—N4—C18—C17	−54.9 (2)
N2—N1—C6—C1	77.3 (3)	N3—C17—C18—N4	53.1 (2)
C13—N1—C6—C1	−114.1 (2)	C21—N4—C19—C20	−128.6 (2)
C12—C7—C8—C9	−1.1 (3)	C18—N4—C19—C20	56.3 (3)
C7—C8—C9—C10	−0.2 (4)	C16—N3—C20—C19	−129.4 (2)
C7—C8—C9—Cl1	179.84 (18)	C17—N3—C20—C19	54.4 (2)
C8—C9—C10—C11	0.6 (3)	N4—C19—C20—N3	−53.7 (2)
Cl1—C9—C10—C11	−179.42 (17)	C19—N4—C21—O2	13.6 (3)
C9—C10—C11—C12	0.3 (3)	C18—N4—C21—O2	−172.0 (2)
C10—C11—C12—C7	−1.5 (3)	C19—N4—C21—N5	−166.6 (2)
C10—C11—C12—C13	−179.7 (2)	C18—N4—C21—N5	7.9 (3)
C8—C7—C12—C11	1.9 (3)	C22—N5—C21—O2	11.2 (3)
C8—C7—C12—C13	−179.9 (2)	C22—N5—C21—N4	−168.7 (2)
N2—N1—C13—C14	0.7 (2)	C21—N5—C22—C23	−164.2 (2)
C6—N1—C13—C14	−168.42 (19)	C21—N5—C22—C27	18.1 (3)
N2—N1—C13—C12	−176.73 (18)	C27—C22—C23—C24	−2.4 (4)
C6—N1—C13—C12	14.1 (3)	N5—C22—C23—C24	179.8 (2)
C11—C12—C13—N1	−148.1 (2)	C22—C23—C24—C25	0.9 (4)
C7—C12—C13—N1	33.7 (3)	C23—C24—C25—C26	1.2 (4)
C11—C12—C13—C14	35.1 (3)	C23—C24—C25—C28	−175.3 (2)
C7—C12—C13—C14	−143.0 (2)	C24—C25—C26—C27	−1.8 (4)
N1—C13—C14—C15	−0.6 (2)	C28—C25—C26—C27	174.8 (2)
C12—C13—C14—C15	176.6 (2)	C25—C26—C27—C22	0.2 (4)
N1—N2—C15—C14	0.0 (2)	C23—C22—C27—C26	1.8 (3)
N1—N2—C15—C16	175.01 (17)	N5—C22—C27—C26	179.4 (2)

### Hydrogen-bond geometry (Å, °)

**Table:**

*D*—H···*A*	*D*—H	H···*A*	*D*···*A*	*D*—H···*A*
N5—H1N5···O1^i^	0.87 (3)	2.14 (3)	2.958 (3)	157 (2)
C2—H2A···N2^ii^	0.93	2.49	3.386 (3)	161
C4—H4A···O1^iii^	0.93	2.42	3.310 (3)	161
C7—H7A···O2^iv^	0.93	2.54	3.312 (3)	140

Symmetry codes: (i) *x*, −*y*+1/2, *z*−1/2; (ii) *x*, −*y*+1/2, *z*+1/2; (iii) *x*−1, *y*, *z*; (iv) *x*, *y*, *z*+1.
